# Computerized Casts for Orthodontic Purpose Using Powder-Free Intraoral Scanners: Accuracy, Execution Time, and Patient Feedback

**DOI:** 10.1155/2018/4103232

**Published:** 2018-04-23

**Authors:** Maria Francesca Sfondrini, Paola Gandini, Maurizio Malfatto, Francesco Di Corato, Federico Trovati, Andrea Scribante

**Affiliations:** Unit of Orthodontics and Paediatric Dentistry, Section of Dentistry, Department of Clinical, Surgical, Diagnostic and Paediatric Sciences, University of Pavia, Pavia, Italy

## Abstract

**Introduction:**

Intraoral scanners allow direct images of oral situation, with fewer steps than conventional impressions. The purpose of this study was to compare the accuracy of digital impressions, traditional impressions, and digitalization of full-arch gypsum models, to evaluate timing of different methods and finally to study perception of patients about conventional and digital impression techniques.

**Methods:**

Dental arches of fourteen patients were evaluated by alginate impression, titanium dioxide powder-free intraoral scanning (Trios, 3Shape), and digitalization obtained from gypsum models using the same scanner. Conventional and digital techniques were evaluated through measurements (lower and upper arch anteroposterior length, lower and upper intercanine distance, and lower and upper intermolar distance) with a caliber for analogic models and using a computer software for digital models (Ortho Analyzer, Great Lakes Orthodontics). In addition, chairside and processing times were recorded. Finally, each patient completed a VAS questionnaire to evaluate comfort. Statistical analyses were performed with ANOVA and Tukey tests for accuracy measurements and paired *t*-test for times and VAS scores. Significance was predetermined at *P* < 0.05.

**Results:**

The measurements obtained with intraoral scanning, gypsum models after conventional impression, and digitalized gypsum models were not significantly different. Both chairside and processing times of digital scanning were shorter than the traditional method. VAS reporting patients comfort were significantly higher when evaluating digital impression.

**Conclusions:**

Intraoral scanners used for orthodontic applications provide useful data in clinical practice, comparable to conventional impression. This technology is more time efficient than traditional impression and comfortable for patients. Further evolution with more accurate and faster scanners could in future replace traditional impression methods.

## 1. Introduction

The use of intraoral scanners is a growing phenomenon involving many areas of dentistry. Several studies in literature showed the accuracy of this method, investigating single elements or arch's portions commonly used in restorative or prosthodontic dentistry, such as crowns, inlays, onlays, and bridges [1–4].

The use of digital casts has been proposed also for full-arch approach, thus allowing use for orthodontic purposes, such as for diagnosis, appliance fabrication, and outcome evaluation [5, 6]. Previous authors evaluated the validity of alginate impressions [7] and digitalization of gypsum or plaster models [8]. Recently, full-arch scan directly on patient's mouth has been proposed during different phases of orthodontic treatment [9].

The use of the intraoral scanner allows the operator to work immediately on the image obtained by completely eliminating the cleansing, disinfection, and casting steps of the traditional alginate method [10]. Additionally, the time spent on packing and sending models to the lab is claimed to be greatly reduced because it is possible to send immediately the file obtained through scanning. Therefore, the development of the scanners could potentially replace in the future either alginate impressions and digitization of gypsum models. In fact, intraoral scanners nowadays present lower shift values for lines and angles measurements than the conventional impression technique followed by indirect digitalization [11, 12] and similar clinical precision compared with conventional impressions [1, 13].

However, new technologies have to be extensively tested in order to be accepted to replace traditional methods. At the present time, there are a small number of studies in literature about the accuracy of measurements on digital full arches obtained with intraoral scanners, as well as the amount of data pertaining to scan time and patient satisfaction index [5]. Moreover, to our knowledge, there is no scientific research that evaluated a powder-free digital impression method for orthodontic purposes.

On the basis of these considerations, the objective of the present study was to evaluate

(1) the accuracy of analog measurement of gypsum casts compared with virtual 3D models obtained by direct and indirect data capturing with a powder-free intraoral scanner,

(2) the time efficiency of the complete workflow of conventional and digital impressions methods,

(3) the patient's comfort during conventional and digital impressions acquisitions.

 The null hypothesis of the study was that there is no significant difference in measurement accuracy among gypsum cast, digitalized gypsum, and intraoral scanning. Moreover, for time efficiency and patient's comfort, the null hypothesis was that there is no significant difference between conventional and digital impressions.

## 2. Materials and Methods

The present crossover study was conducted at the Unit of Orthodontics and Paediatric Dentistry, Section of Dentistry, Department of Clinical, Surgical, Diagnostic and Paediatric Sciences, University of Pavia, Pavia, Italy. The present experimentation followed Helsinki Declaration. Internal Unit Review Board accepted the study design (Ref: 17-0317).

14 consecutive patients were enrolled (4 male and 10 female with a mean age of 20.4 years). Inclusion criteria of this study were full permanent dentition, no remaining deciduous teeth, no impacted teeth, and no supernumerary teeth; the exclusion criteria chosen included history of mental or developmental disabilities, craniofacial anomalies, epilepsy, dental fear, and hyperactive gag reflex. Informed consent was received from all subjects. Detailed information on the research purposes, expected duration of the study, and study protocols were explained.

Each participant in the study was subjected to alginate impression and intraoral scanning. In addition, chairside timing, processing timing, and responses of each individual patient to compliance questionnaires were evaluated for both impression methods used.

Alginate impressions were taken with irreversible hydrocolloid Jeltrate Fast set (Dentsply GAC, Bohemia, USA). The impression was taken according to the current guidelines [7], and the patients were informed earlier about the positions to keep during the procedure. All the impressions were checked by the same operator and, once the diagnostic validity was verified, they were cleansed, disinfected, and stored in a damp environment to avoid deformation, according to the manufacturer's instructions. Additionally, a wax bite was registered for each patient (Tenatex Red, Kemdent, Swindon, UK). The wax was prepared in triple layer and then it was adapted from a standard size to the upper arch shape of the patient, height 2.5 mm. Bite registration was taken with the patient's teeth in centric occlusion. Subsequently, alginate impressions were cast with gypsum Kerr type IV.

Intraoral scan was performed using a powder-free intraoral digital monochromatic scanner (Trios 3 Mono Intraoral Scanner, 3Shape, Copenhagen, Denmark) according to the manufacturer's guidelines for scanning strategy. Each patient was instructed about the positions to keep and about all the steps provided. Each sextant was treated individually, recording the vestibular, lingual, and occlusal surface of each element and paying particular attention to the control of soft tissue and tongue. The patient was seated upright, and moisture control was obtained with cheek retractors and absorbent triangles. The dental assistant removed excess of saliva with air suction unit and distanced soft tissues from teeth with an intraoral mirror. The teeth were then scanned starting in the mandibular right quadrant and ending in the maxillary left quadrant. The second and third molars were included in the scan, when present. The bite registration was obtained by scanning the buccal surfaces of the patient's right premolars and first molars in centric occlusion. At the end of each recording, the scanner was stopped, allowing the operator to check the validity. When the lower arch was completed, the upper arch was recorded, starting from the first sextant. Once the scanning of both arches has been completed, the patient was asked to achieve the usual occlusion with closed lips. After controlling the correct position, a bite registration was obtained by scanning vestibular position of all dental surfaces exposed. Scans of gypsum models were taken with the same intraoral monochromatic scanner (Trios 3 Mono Intraoral Scanner, 3Shape, Copenhagen, Denmark).

Conventional and digital impressions were taken in the same appointment by the same trained operator. Subsequently, the patients were asked to fill out a questionnaire about the two methods. The survey was structured in five questions about overall impression, comfort, timing, instrument size, and gag reflex linked to the two different impression methods. Each question of the survey was accompanied by a 10 cm visual analog scale (VAS) to note perceived comfort during both procedures. The value “10” represented maximum comfort (no discomfort at all), whereas the value “0” meant the maximum discomfort (maximum complaint).

In order to analyze the difference in accuracy among analog model, intraoral digital model, and digitalization of the gypsum model, on each model points of repetition were highlighted, as upper and lower interincisors point, canines' cusps, central pits of first molars, and mesiolingual cusps of upper first molars. Six different dentoalveolar measurements were evaluated: lower arch anteroposterior length, upper arch anteroposterior length, lower intercanine distance, upper intercanine distance, lower intermolar distance, and upper intermolar distance.

**Figure 1:**
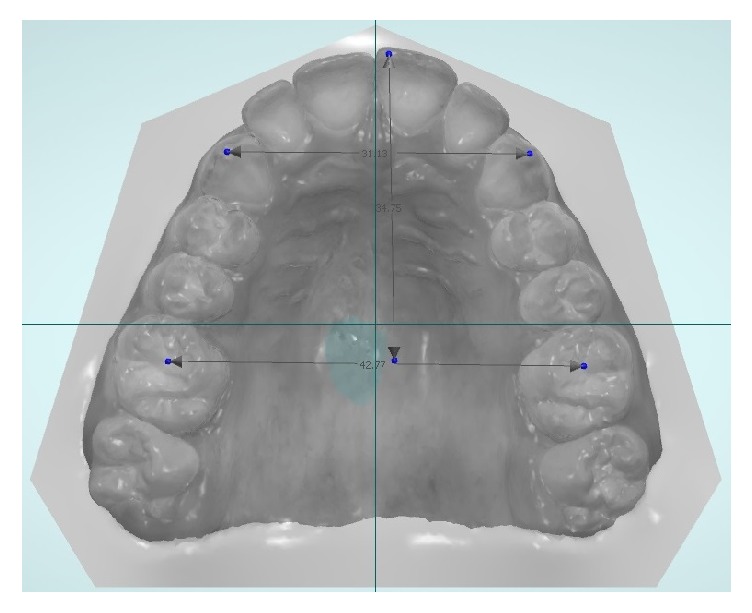
Measurement points for upper arch.

**Figure 2:**
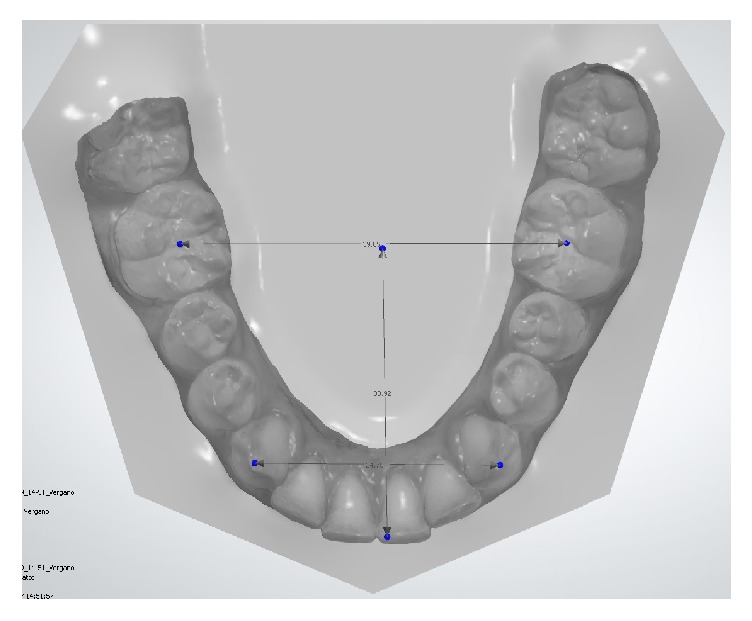
Measurement points for lower arch.

A caliber (Fasa Group, Lauf, Germany) was used to evaluate analog measurement of gypsum casts. For digital upper (Figure 1) and lower (Figure 2) models, the measurements were carried out using a computer software (Ortho Analyzer, Great Lakes Orthodontics, Tonawanda, USA).

In relation to the timing needed for the impressions, a synchronized stopwatch was used for the analogic method in each of the steps carried out: time 1, which included time for both alginate impressions (including tray size choice and alginate mixing) and wax bite registration (including heating); time 2, for cleaning, disinfection (water washing and disinfectant spray application), and packaging (for laboratory selling); time 3 for realization of gypsum models (impressions cast with gypsum). The three times were then added to obtain the timing required for traditional impression method. In digital method, the scanning times of all single sextants and the timing for occlusion registration indicated by the software were considered. In addition, the time needed for saving and sending data to the laboratory was calculated. The time spent talking to patients explaining procedures and time for rinsing after impression were not reported for both methods.

Finally, the last purpose of the report was the evaluation of comfort of the patients. VAS scores of the questionnaires delivered during the trial were analyzed.

**Figure 3:**
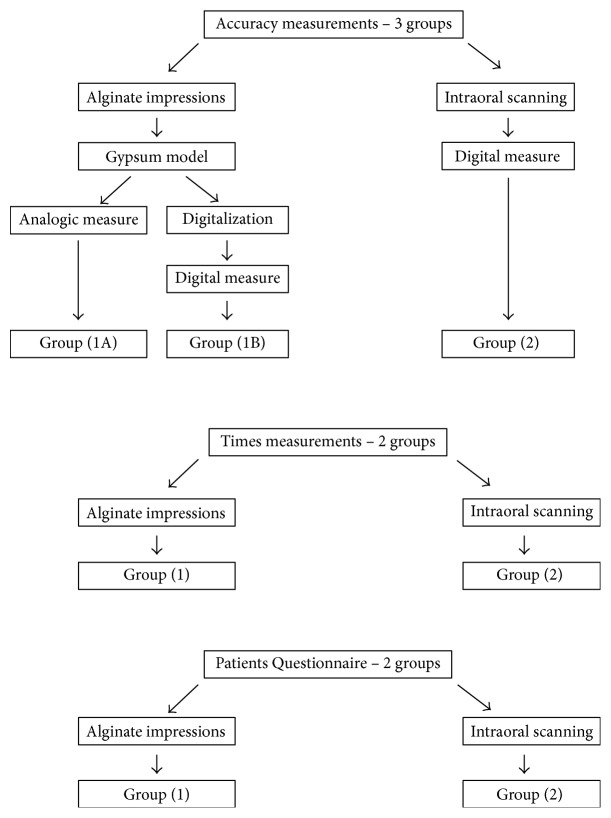
Flow chart with diagrammatic representation of the test groupings for the three variables.

Therefore, the experimental groups for measurement analysis were divided as follows (Figure 3):

(1A) Analogic evaluation of the gypsum cast
(1B) Computerized evaluation of the virtual model after indirect digitalization of gypsum cast
(2) Computerized evaluation of the virtual model after direct digitalization by an intraoral scanner.

On the other hand, the research groups for times and patients feedback were

1. conventional impression,
2. digital impression.

Statistical analysis was performed with a computer software (R version 3.1.3, R Development Core Team, R Foundation for Statistical Computing, Wien, Austria). Sample size calculation was performed. To estimate the method error, the same operator retraced analogic and digital measures after a period of 4 weeks [14] and results were tested with *t*-test; no significant variations were reported.

Mean, standard deviation, minimum, median, and maximum were chosen for descriptive statistics and were calculated for the various groups. Kolmogorov-Smirnov test assessed Gaussian data distribution for all variables. ANOVA (analysis of variance) and Tukey tests were applied for accuracy measurements and paired *t*-test was for times and VAS scores.

Significance was predetermined at *P* < 0.05 for all statistical tests.

## 3. Results

**Figure 4:**
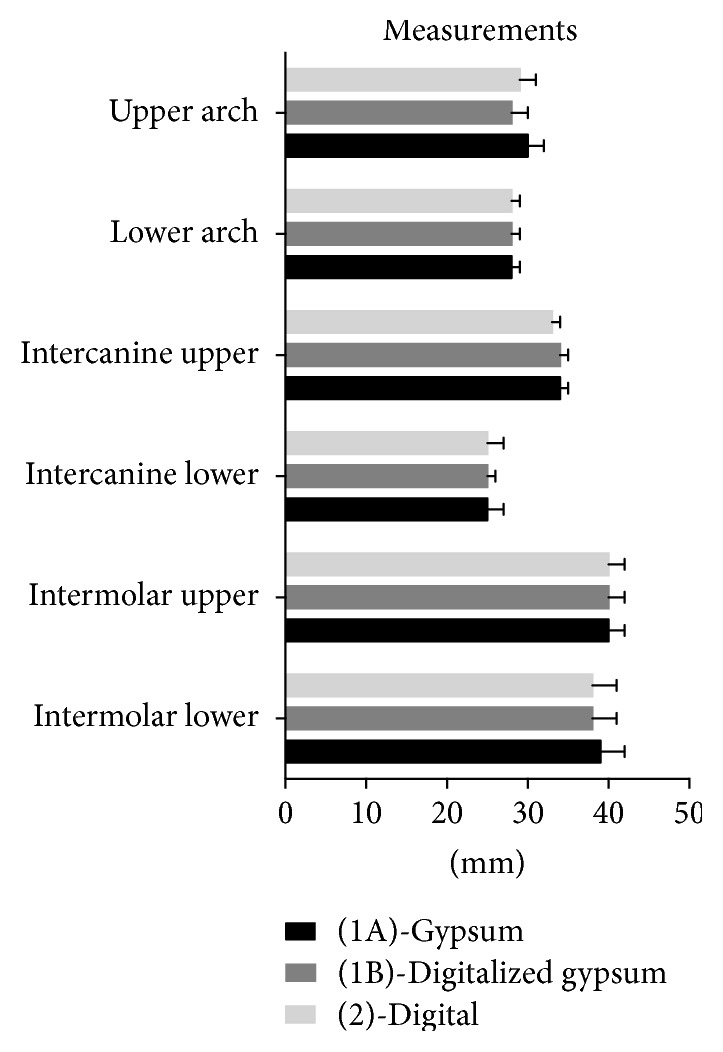
Accuracy of measurements (mm) among gypsum, digitalized gypsum, and full digital models.

The evaluation of the difference in accuracy between the conventional and digital methods was made by comparing 6 types of dentoalveolar measurements previously expressed. The results are showed in Figure 4. Descriptive statistics included mean, standard deviation, minimum, median, and maximum values for each group (Table 1). No significant difference was reported among various groups (*P* > 0.05).

**Figure 5:**
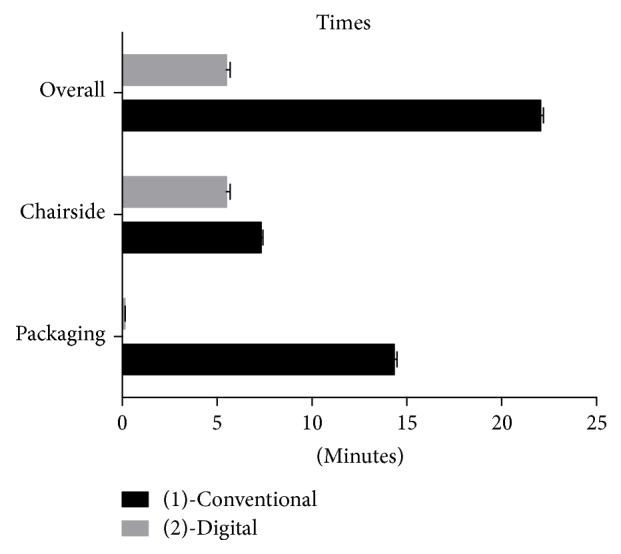
Time measurements (minutes) of conventional and digital techniques.

The time requirements for alginate impressions and intraoral scans are reported in Table 2. As showed in Figure 5, the scan method showed chairside and processing times significantly shorter than those required to perform alginate impressions (*P* < 0.0001).

**Figure 6:**
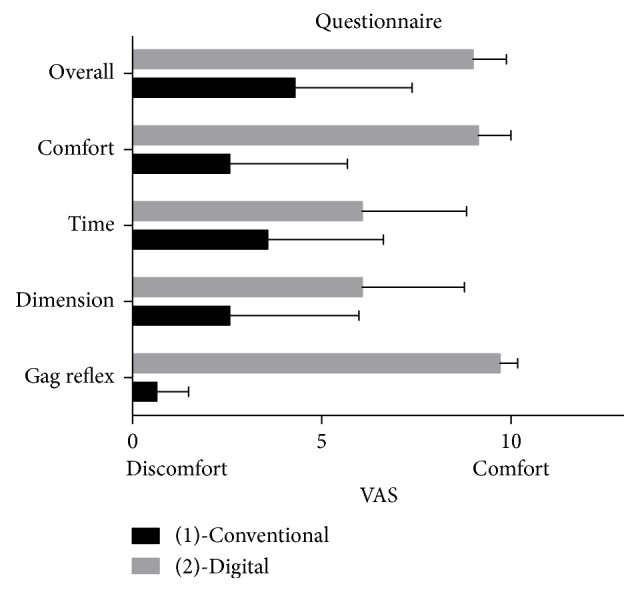
Questionnaire results for conventional and digital techniques (VAS scores: 0: maximum discomfort; 10: maximum comfort).

Patients feedback was evaluated with a 5-point VAS questionnaire (Figure 6). All the patients enrolled in the study provided a full compilation of the survey. As showed in Table 3 patient comfort was significantly higher with digital impression system than with conventional alginate.

## 4. Discussion

The null hypothesis of the present report was accepted for accuracy measurements analysis and rejected when evaluating execution times and patient's feedback. A new technology, such as intraoral scanning, has to demonstrate excellent results about accuracy, timing, and patient satisfaction in order to be used in orthodontic daily practice. When evaluating accuracy of the measurements, no significant differences were reported among conventional alginate impressions, digital cast measurements, and digitalized gypsum models. In the present report, it was decided to perform both alginate and digital impressions in the same session in order to have immediate patient feedback and to obtain a cross validation of each variable tested. 14 consecutive patients were enrolled. This number is comparable with those of other authors in literature, focusing on the efficacy of intraoral scanner [15, 16].

The precision of intraoral digital scans has been evaluated in several studies. Positive results regarding the high accuracy of digital models by intraoral scanning have been reported [17]. However, in many studies the evaluation was conducted on single dental elements, thus reporting the accuracy of the direct digital models in accordance with restorative dentistry objectives [18, 19]. Other reports studied intraoral scanners for full-arch evaluation and demonstrated that intraoral scanners are clinically acceptable for diagnosis and treatment planning [9, 15, 20, 21]. Some authors measured some slight errors in the position of dental elements ranging from −0.05 to 0.21 mm and imprecisions for arch length and width from −0.07 to 0.17 mm. However, the study concluded that the digital models of the entire dental arches could have clinical validity in orthodontics [22]. In fact it has to be specified that data loss is possible in the digital method, and the accuracy of a digital model can be limited by the resolution of the scanner. In the present study a scanner with 50-micron accuracy has been used [23]. Furthermore, even if accuracy of intraoral scanners has been reported to be high, during the finding of key points on teeth, a slight variability in point detection could happen. However, in the present study, the method error analysis showed no significant difference between measures taken on the same patients immediately after scanning and after one month; thus digital point detection could be considered repeatable. Moreover, the lack of significant differences among intraoral digital measures versus direct and indirect gypsum measures means that the tested intraoral scanner presents reliability of the scanned data if compared with conventional analogic gypsum measures.

The second variable evaluated in the present report was execution time of the two impression methods. During clinical practice, the time required to perform impressions can clearly play an important role, as orthodontists could need many casts during treatment. In this study, it has been showed that the total time of an alginate impression is significantly higher than the time required for a powder-free intraoral digital scan. The time difference is related to lower chair time but is mainly explained with packaging and processing times that are strongly lowered with digital shipping. This time reduction is not confirmed by previous authors that evaluated other intraoral scanners that need application of titanium dioxide powder [5]. In fact the use of powder could elongate acquisition time. In the present report, a powder-free system has been tested and this is the reason that presumably explains difference between the results. No other time-studies have been conducted with powder-free systems. However, some authors pointed out that the time needed to perform digital impression decreases with the increase in clinical experience also for powder-activated scanners [24, 25].

The last variable evaluated in the present report is patient comfort. Previous studies showed a tendency of patients to prefer traditional impressions to digital intraoral scans [15], mostly because the conventional method is easier and faster than the digital method. However, recent studies have demonstrated a reversal of pathways by young patients with a preference of the digital method [5]. This is in agreement with the present report in which patients showed a clear preference for digital intraoral scanning, associated with a condition of greater comfort. Even with regard to gag reflex, all patients preferred the choice of the digital method. VAS were significantly higher along all the questions, even if the differences are smaller for the question related to impression times and dimension of instruments used. Presumably many patients considered the time of intraoral scan still too long and the dimension of the digital armamentarium too large. In fact some areas are difficult to reach with intraoral scanner, such as lower posterior lingual sectors. Moreover during occlusal recording, the mesiodistal passage of the scanner could cause discomfort in the posterior region for contact with the front edge of the mandible and for the presence of the natural contraction of the masseter muscle. Manufacturers are increasing their efforts to offer speeder and smaller intraoral scanners. Technology and hardware improvements could in future reduce this problem. However, in the present questionnaire results, overall opinion is significantly favorable to intraoral scanner that is considered more comfortable than conventional impression method.

The switch to a new technology in routine practice depends on many factors, such as the level of acceptance of the patients, simplicity of use, and acceptable costs. As for other advances that have been proposed in the near past to improve efficiency of orthodontic treatment, such as high output curing lights [26], movement speed enhancers [27], adhesive precoated brackets [28], no-primer adhesives [29], indirect bonding technique [30], self-ligating brackets [31], and smile design software [32], nowadays also for digital impression system an experimental phase is beginning to know merits and defects of the method. Orthodontic practitioners will consider intraoral scanning technology only if it proves to be accurate, efficient, and convenient for both clinician and patient. The present report showed that the digital intraoral scanner tested has similar accuracy, lower times, and higher comfort if compared to conventional impressions. Moreover, intraoral digital scanning reduces the steps in the acquisition of the models, and it allows the clinician to send digitally the data obtained directly to the dental laboratory. On the other hand the cost of digital impression systems is still very expensive. Moreover, further studies are needed, as results obtained are often influenced by the operator's experience [33]. However, the advantages of digital system over conventional method, together with the increase in performance and the cost lowering of scanners, could imply in future an ever greater transition from the traditional method of impressions to intraoral scanning technology in orthodontic daily practice.

## 5. Conclusions

The results of this study confirm the potential of intraoral scanning to acquire data as accurate as alginate impressions for orthodontic applications. Intraoral scan can produce digital models useful in clinical practice for diagnosis, treatment planning, and documentation of treatment outcomes. New generation powder-free scanner also reduces both chairside and processing times if compared to alginate impressions. Finally, patients expressed greater comfort and lower gag reflex using the intraoral scanner.

It can be argued that digital intraoral scanning technology can be used as a reliable alternative to traditional method for orthodontic purposes, even if further studies are needed to analyze also other features of these devices.

## Figures and Tables

**Table 1:** Descriptive statistics of accuracy (mm) of three different measurements (conventional measurement, digital measurement, and digitalization of gypsum model).

Measurements	Group	Model	Mean	SD	Min	Median	Max	Significance
Upper arch	(1A)	Gypsum	30.35	2.28	24.8	31	32.5	
	(1B)	Digitalized gypsum	28.9	2.72	21.72	29.43	32.25	ns
	(2)	Digital	29.46	2.66	22.66	29.9	33.18	

Lower arch	(1A)	Gypsum	28.06	1.63	24.5	28.65	30	
	(1B)	Digital gypsum	28.07	1.39	25.09	28.15	30.33	ns
	(2)	Digital	28	1.30	25.23	28.2	29.99	

Intercanine upper	(1A)	Gypsum	34.03	1.83	31.4	33.7	37.6	
	(1B)	Digital gypsum	34	1.84	30.77	33.54	37.46	ns
	(2)	Digital	33.8	1.93	31.15	33.54	36.76	

Intercanine lower	(1A)	Gypsum	25.87	2.15	22	25.63	31	
	(1B)	Digital gypsum	25.96	1.99	21.54	25.61	29.95	ns
	(2)	Digital	25.64	2.01	21.98	25.51	30.05	

Intermolar upper	(1A)	Gypsum	40.78	2.12	36.9	40.85	45.5	
	(1B)	Digital gypsum	40.53	2.40	36.98	40.1	46.25	ns
	(2)	Digital	40.36	2.24	37.37	40.04	46.11	

Intermolar lower	(1A)	Gypsum	39.05	3.48	31	39.55	45.2	
	(1B)	Digital gypsum	38.93	3.36	31.66	39.88	45.65	ns
	(1B)	Digital gypsum	38.93	3.36	31.66	39.88	45.65	

**Table 2:** Descriptive statistics of timing (minutes : seconds) related to conventional and digital impression methods.

Times	Group	Impression method	Mean	St Dev	Min	Median	Max	Significance
Total time	(1)	Conventional	22:06	00:15	21:45	22:03	22:35	*P* < 0.0001
	(2)	Digital	05:49	00:20	05:15	05:54	06:14	
Chairside time	(1)	Conventional	07:32	00:10	07:10	07:32	07:50	*P* < 0.0001
	(2)	Digital	05:49	00:20	05:15	05:54	06:14	
Processing time	(1)	Conventional	14:34	00:14	14:15	14:29	15:02	*P* < 0.0001
	(2)	Digital	00:14	00:01	00:13	00:15	00:17	

**Table 3:** Questionnaire results. VAS scale ranged from 0 (maximum discomfort) to 10 (no discomfort at all).

Question	Group	Technique	Mean	SD	Min	Median	Max	Significance
What was the overall sensation about the impression technique?	(1)	Conventional	4.29	3.10	0.00	3.50	9.00	*P* < 0.001
	(2)	Digital	9.00	0.88	7.00	9.00	10.00	
Did you find the procedure comfortable?	(1)	Conventional	2.57	3.11	0.00	1.50	9.00	*P* < 0.0001
	(2)	Digital	9.14	0.86	7.00	9.00	10.00	
How do you consider the time to complete the procedure?	(1)	Conventional	3.57	3.06	0.00	2.50	9.00	*P* < 0.05
	(2)	Digital	6.07	2.76	1.00	6.50	10.00	
How do you consider the dimension of instruments used?	(1)	Conventional	2.57	3.41	0.00	0.50	9.00	*P* < 0.01
	(2)	Digital	6.07	2.70	2.00	6.50	10.00	
When evaluating gag reflex was the procedure comfortable?	(1)	Conventional	0.64	0.84	0.00	0.00	2.00	*P* < 0.0001
	(2)	Digital	9.71	0.47	9.00	10.00	10.00	
